# Identification of MicroRNAs Involved in Growth Arrest and Apoptosis in Hydrogen Peroxide-Treated Human Hepatocellular Carcinoma Cell Line HepG2

**DOI:** 10.1155/2016/7530853

**Published:** 2016-08-15

**Authors:** Yuan Luo, Xinyu Wen, Ling Wang, Jing Gao, Zi Wang, Chunyan Zhang, Pengjun Zhang, Chengrong Lu, Lianning Duan, Yaping Tian

**Affiliations:** ^1^Core Laboratory of Translational Medicine, State Key Laboratory of Kidney Disease, Chinese PLA General Hospital, Beijing 100853, China; ^2^Aviation Medicine Research Laboratory, Air Force General Hospital, PLA, Beijing 100142, China; ^3^Department of Clinical Biochemistry, Chinese PLA General Hospital, Beijing 100853, China

## Abstract

Although both oxidative stress and microRNAs (miRNAs) play vital roles in physiological and pathological processes, little is known about the interactions between them. In this study, we first described the regulation of H_2_O_2_ in cell viability, proliferation, cycle, and apoptosis of human hepatocellular carcinoma cell line HepG2. Then, miRNAs expression was profiled after H_2_O_2_ treatment. The results showed that high concentration of H_2_O_2_ (600 *μ*M) could decrease cell viability, inhibit cell proliferation, induce cell cycle arrest, and finally promote cell apoptosis. Conversely, no significant effects could be found under treatment with low concentration (30 *μ*M). miRNAs array analysis identified 131 differentially expressed miRNAs (125 were upregulated and 6 were downregulated) and predicted 13504 putative target genes of the deregulated miRNAs. Gene ontology (GO) analysis revealed that the putative target genes were associated with H_2_O_2_-induced cell growth arrest and apoptosis. The subsequent bioinformatics analysis indicated that H_2_O_2_-response pathways, including MAPK signaling pathway, apoptosis, and pathways in cancer and cell cycle, were significantly affected. Overall, these results provided comprehensive information on the biological function of H_2_O_2_ treatment in HepG2 cells. The identification of miRNAs and their putative targets may offer new diagnostic and therapeutic strategies for liver cancer.

## 1. Introduction

Hepatocellular carcinoma (HCC) is the most common primary liver cancer and accounts for about 80% of all cases of this disease. Globally, HCC is the fifth most common cancer, but also the third cause of cancer-related mortality [1, 2]. It is established that various factors are associated with the development of HCC, including hepatitis virus infection, alcoholic liver damage, ingestion of aflatoxin B1, nonalcoholic fatty liver disease, obesity, diabetes mellitus, and iron accumulation. Although the dominant role of these risk factors in different countries or regions might vary, the occurrence and development of HCC are a multistep process associated with years of chronic inflammation of liver [3–5]. Inflammatory cells, including neutrophils, macrophages, mast cells, and dendritic and natural killer cells, are recruited within inflammatory sites to release chemical mediators, such as cytokines, chemokines, and reactive oxygen species (ROS). The latter, in turn, might play a vital pathogenic role in the long-term progression, originating from chronic inflammation to hepatitis and ultimately leading to cancer [6–8].

ROS are chemically reactive molecules containing oxygen. The group includes hydrogen peroxide (H_2_O_2_), superoxide anion (O_2_^−^), hydroxyl radical (OH^•^), singlet oxygen (^1^O_2_), ozone (O_3_), and some other small molecules. In a biological context, ROS are mainly deprived from oxidative metabolism as natural byproducts and play important roles in cell signaling and homeostasis. However, in some pathological conditions or during environmental stress, ROS levels can increase dramatically and lead to the disruption of the prooxidant/antioxidant equilibrium. This is known as oxidative stress and it could lead to damaging of many intracellular molecules, including DNA, RNA, lipids, and proteins [9–11]. Specifically, ROS can promote many aspects of tumor development and progression either directly by activating proinflammatory transcriptional factors such as NF-*κ*B and AP-1 or indirectly by inducing DNA damage and oncogene activation [12]. Meanwhile, some latest studies have cast new light on the complicated interplay network between inflammation, oxidative stress, cancer, and microRNAs (miRNAs) [13, 14]. The latter, which are a series of small noncoding RNAs (containing about 22 nucleotides), could modulate gene expression through canonical base pairing between the seed sequences of themselves and 3′ untranslated region (3′ UTR) of target mRNAs. In general, miRNAs downregulate gene expression by inhibiting the translation and/or reducing the stability of target mRNAs and therefore provide a novel level of posttranscriptional regulation [15, 16].

Although publications in the past decade have described the involvement of miRNAs in almost all kinds of physiological and pathological processes, little is known about the specific mechanisms of the interaction between oxidative stress and miRNAs. To the best of our knowledge, no previous researches have reported the miRNAs expression profile in H_2_O_2_ treated human hepatocellular carcinoma cell lines. Therefore, in this study, we examined the changes of the miRNAs expression profiles in HepG2 cells upon treatment with H_2_O_2_. The target genes of significantly changed miRNAs were predicted by in silico prediction algorithms. Combined with biological experiments, we present a more holistic view indicating that H_2_O_2_-sensitive miRNAs regulate cell proliferation inhibition, cycle arrest, and apoptosis promotion.

## 2. Materials and Methods

### 2.1. Cell Culture

HepG2 cells were obtained from China infrastructure of cell line resources. The cells were maintained as a monolayer culture in Dulbecco's modified Eagle's medium (DMEM; Gibco, Invitrogen Life Technologies, Carlsbad, CA, USA) supplemented with 10% fetal bovine serum (FBS; Gibco, Invitrogen Life Technologies, Carlsbad, CA, USA), 100 IU/mL penicillin, and 100 *μ*g/mL streptomycin. HepG2 cells were cultured in a humidified incubator with 5% CO_2_ at 37°C.

### 2.2. Cell Viability Assay

Cell viability and proliferation were quantified by Cell Counting Kit-8 (CCK-8; Dojindo Molecular Technologies, Inc., Kumamoto, Japan) according to the manufacturer's protocol. To assess the cell viability, cells were seeded into 96-well plates at a concentration of 8 *∗* 10^3^ HepG2 cells/well and each group was repeated in 5 wells. After the cells were treated with various concentrations of H_2_O_2_ for 24 hours (h), 10 *μ*L CCK-8 was added into each well. After mixing, the cells were incubated for an additional 2 h, and then a microplate reader (RT-6000; Rayto, Rayto Life and Analytical Sciences Co., Ltd., Guangdong, China) was used to measure the absorbance at 450 nm (OD450) for each well. The cell viability rates were calculated according to the following formula: the cell viability ratio (%) = [(As − Ab)/(Ac − Ab)]*∗*100%, where As is the OD450 of H_2_O_2_ experiment group, Ab is the OD450 of blank control group, and Ac is the OD450 of non-H_2_O_2_ control group. The cell viability graph was drawn in which the viability ratios were plotted at the vertical axis and H_2_O_2_ concentration was plotted at the horizontal axis. The IC50 (half maximal inhibitory concentration) value, which represents the concentration of H_2_O_2_ in determining 50% of cell viability, was calculated by nonlinear regression analysis using GraphPad Prism software (San Diego, USA).

### 2.3. Cell Proliferation Assay

To assess the cell proliferation, cells were seeded into 96-well plates at a concentration of 2 *∗* 10^3^ HepG2 cells/well and each group was repeated in 5 wells. After cells were treated with 0–100 *μ*M of H_2_O_2_ for 24 h, the growth medium was replaced by fresh medium. At the following 4 detection time points (0, 24, 48, and 72 h), 10 *μ*L CCK-8 was added into each well. After mixing, cells were incubated for an additional 2 h before measuring the OD450. The cell proliferation graph was drawn, with OD450 plotted at the vertical axis and incubation time in hours at the horizontal axis.

### 2.4. Cell Cycle Assay

The cell cycle and apoptosis were analyzed by flow cytometry (FCM). To assess the cell cycle, HepG2 cells in logarithmic phase were harvested and plated in 24-well culture plates at a concentration of 2 *∗* 10^5^ cells/mL. Cells were starved overnight to achieve synchronization and then treated with 0–200 *μ*M of H_2_O_2_ for 24 h. Using FCM instrument (FACS Calibur, BD Biosciences, San Jose, CA, USA), cell cycle was detected with cycle test Plus DNA reagent kit (BD Biosciences, San Jose, CA, USA) according to the manufacturer's instructions.

### 2.5. Cell Apoptosis Assay

To assess the level of apoptosis, HepG2 cells were plated in 24-well culture plates in the same concentration as mentioned above. After cell adhering, 0–800 *μ*M of H_2_O_2_ were added and cells were incubated for 24 h. The Annexin V-PE/7-AAD Apoptosis Detection Kit (BD Biosciences, San Jose, CA, USA) was used to detect cell apoptosis according to the manufacturer's instructions.

### 2.6. Determination of Total ROS

Intracellular ROS levels were also determined by FCM. HepG2 cells were plated in 12-well culture plates at a concentration of 2 *∗* 10^5^ HepG2 cells/well. After cell adhering, 0–600 *μ*M of H_2_O_2_ was added and cells were incubated for 24 h. Cells were harvested and then incubated with 10 *μ*M 5-(and-6)-chloromethyl-2′,7′-dichlorodihydrofluorescein diacetate, acetyl ester (CM-DCHF-DA, Invitrogen Life Technologies, Carlsbad, CA, USA), which could be cleaved by intracellular esterases and transformed into a fluorescent dye when oxidized at 37°C for 30 min. The samples were analyzed by FCM instrument with CellQuest software. For each sample, 10,000 cells were analyzed.

### 2.7. RNA Preparation and miRNAs Microarray

HepG2 cells were seeded in 10 cm dishes and cultured as usual until they reached 80% confluence. The cells were treated with H_2_O_2_ (0, 30, and 600 *μ*M) for 24 h and each concentration was repeated in 3 dishes. Total RNA was extracted from the treated cells by using Trizol reagent (Invitrogen Life Technologies, Carlsbad, CA, USA) and then purified with a QIAGEN RNeasy Mini Kit (Qiagen, Valencia, CA, USA). After assessing the RNA's quality and quantity, the miRNAs microarray analysis (Affymetrix microRNA 4.0 Array, Santa Clara, CA, USA) was performed according to the manufacturer's instructions. Briefly, 1 *μ*g of total RNA was labeled with Biotin using the FlashTag*™* Biotin HSR RNA Labeling Kit (Genisphere, Hatfield, PA, USA) and then hybridized overnight with the array, which was washed, stained, and read by an GeneChip Scanner 3000 7G (Affymetrix).

### 2.8. Data Analysis of miRNAs Microarray

CEL-files of the raw data were first exported by Affymetrix GeneChip Command Console Software Version 4.0 (Affymetrix) and then uploaded to the website of Gminix-Cloud Biotechnology Information (GCBI, http://www.gcbi.com.cn/gclib/html/index, Genminix Informatics Co., Ltd., Shanghai, China) for further analysis, including difference analysis of miRNAs profiles, prediction of miRNAs target genes, GO/pathway enrichment analysis, and miRNAs-gene-network and miRNAs-GO-network analysis. The miRNAs array data discussed in this paper has been uploaded to the NCBI Gene Expression Omnibus and is accessible through GEO series accession number GSE84406 (http://www.ncbi.nlm.nih.gov/geo/query/acc.cgi?acc=GSE84406).

### 2.9. Statistical Analysis

Each experiment was performed independently at least 3 times with similar results. Student's *t*-test (two-tailed) was used to determine the statistical significance of quantitative data and Chi-square test was used for the statistical analysis of constituent ratio. *P* < 0.05 was considered to be statistically significant.

## 3. Results

### 3.1. H_2_O_2_ Treatment Decreases Cell Viability and Inhibits Proliferation of HepG2 Cells

**Figure 1:**
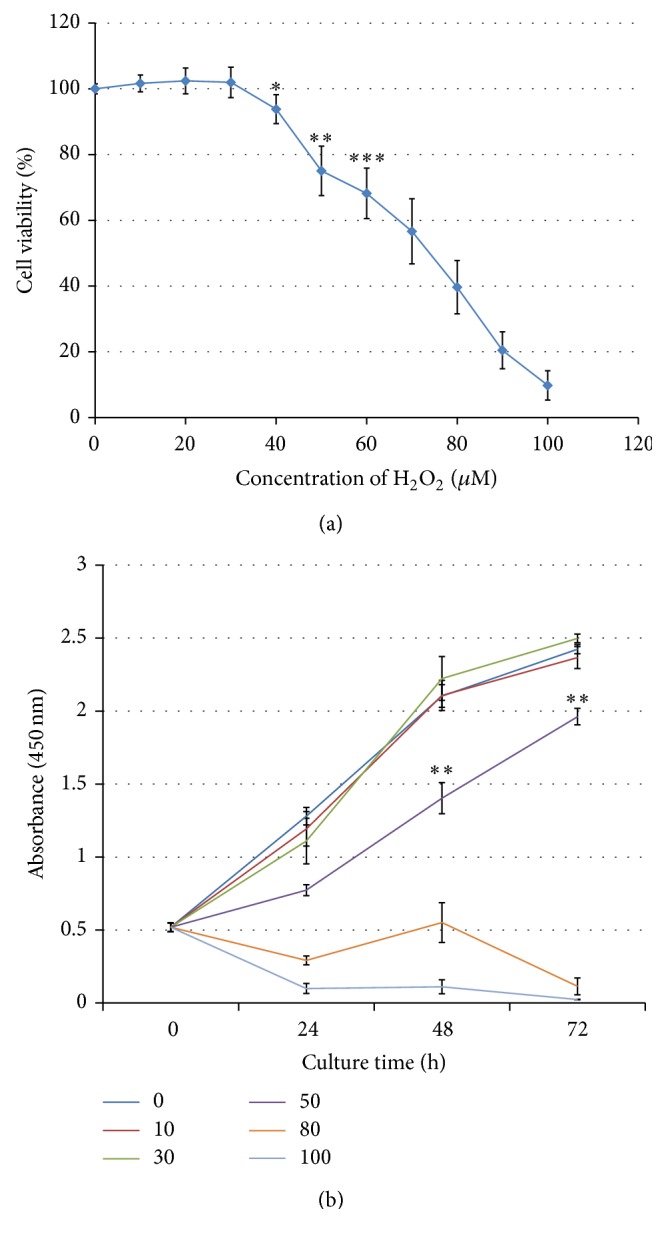
H_2_O_2_ treatment decreases cell viability and inhibits cell proliferation of HepG2 cells. (a) HepG2 cell viability was measured by CCK-8 assay after H_2_O_2_ treatment for 24 h. (b) After H_2_O_2_ treatment for 24 h, the cell proliferation was quantified at different detection periods. Error bars denote mean ± SD. ^*∗*^ *P* < 0.05; ^*∗∗*^ *P* < 0.01; ^*∗∗∗*^ *P* < 0.001.

To determine the cytotoxicity of H_2_O_2_, we comprehensively detected the changes of viability, proliferation, cell cycle, and apoptosis in HepG2 cells. After exposure of HepG2 cells to H_2_O_2_ for 24 h, CCK-8 assay was firstly performed to determine the cell viability and proliferation. As shown in Figure 1, although 30 *μ*M H_2_O_2_ seems to increase the cell viability and even proliferation, there were no statistically significant differences between them and control groups (*P* > 0.05). Conversely, in the presence of higher concentrations of H_2_O_2_, both cell viability and proliferation decreased significantly (*P* < 0.05). The IC50 value calculated through nonlinear regression analysis was 70.3 *μ*M. These data suggest that H_2_O_2_ could cause an obvious dose-dependent reduction of cell viability and proliferation, while no significant effect could be found at low concentrations of this compound.

### 3.2. H_2_O_2_ Treatment Induces Cell Cycle Arrest and Apoptosis of HepG2 Cells

**Figure 2:**
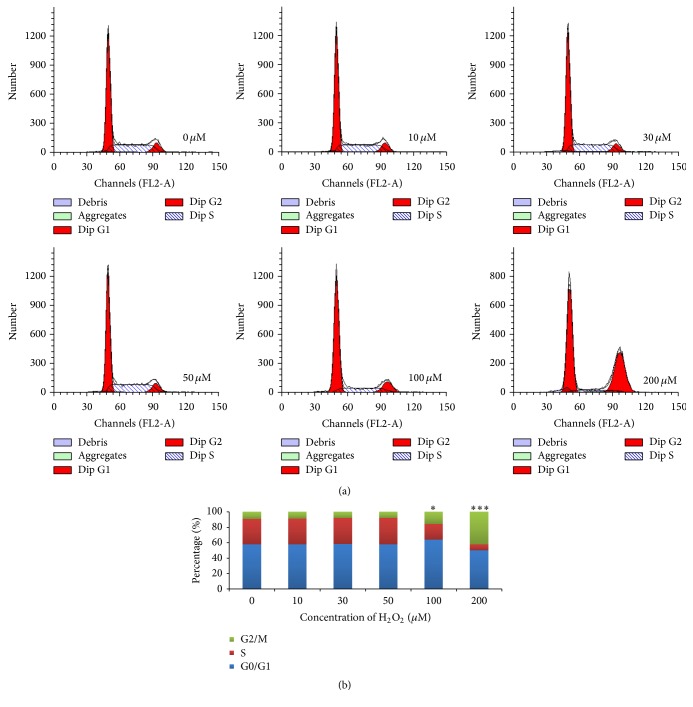
H_2_O_2_ treatment induces cell cycle arrest in G2/M phase. (a) The cell cycle was analyzed by flow cytometry. After synchronization induced by serum starvation overnight, HepG2 cells were treated with H_2_O_2_ for 24 h. (b) The histogram shows the cell cycle percentage detected by FCM. ^*∗*^ *P* < 0.05; ^*∗∗∗*^ *P* < 0.001.

**Figure 3:**
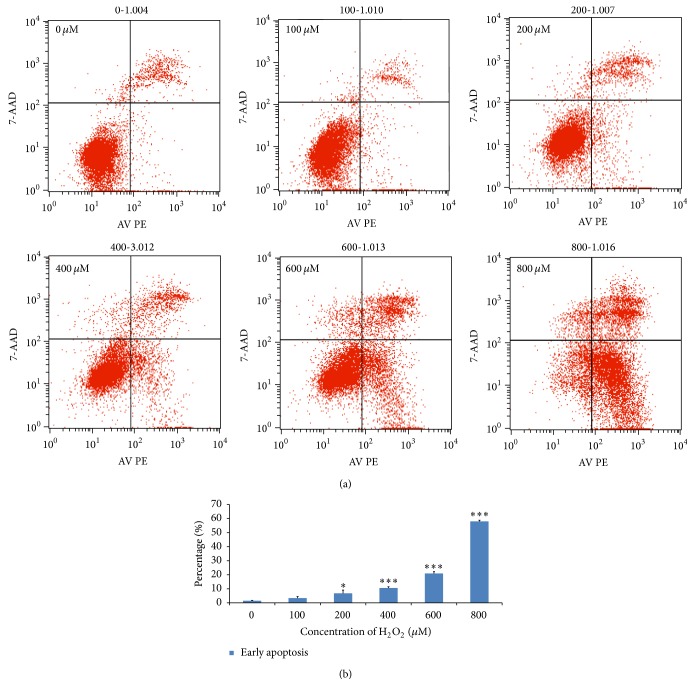
H_2_O_2_ treatment induces cell apoptosis in a dose-dependent manner. (a) The cell apoptosis was analyzed by FCM assay. HepG2 cells were treated with H_2_O_2_ for 24 h. (b) The histogram shows the early apoptotic cell percentage detected by FCM. ^*∗*^ *P* < 0.05; ^*∗∗∗*^ *P* < 0.001.

FCM was utilized to detect the cell cycle and apoptosis of HepG2 cells after H_2_O_2_ treatment. As shown in Figure 2, lower concentrations of H_2_O_2_ did not affect the cell cycle in HepG2 cells (*P* > 0.05). However, once the concentration of H_2_O_2_ was higher than 100 *μ*M, HepG2 cells exhibited significant increase in G2/M phase but obvious reduction in S phase and even G0/G1 phase (*P* < 0.05).  Figure 3 showed that H_2_O_2_ could cause a dose-dependent increase of cell apoptosis, especially early apoptosis. In the presence of 600 and 800 *μ*M H_2_O_2_, a significant (*P* < 0.001) increase in cell early apoptosis of 20.9% and 58.0%, respectively, was observed. These data suggested that H_2_O_2_ could induce cell cycle arrest in G2/M phase and promote a dose-dependent cell apoptosis of HepG2 cells, which might directly cause the reduction of cell viability and proliferation.

### 3.3. H_2_O_2_ Treatment Stimulates ROS in a Dose-Dependent Manner

**Figure 4:**
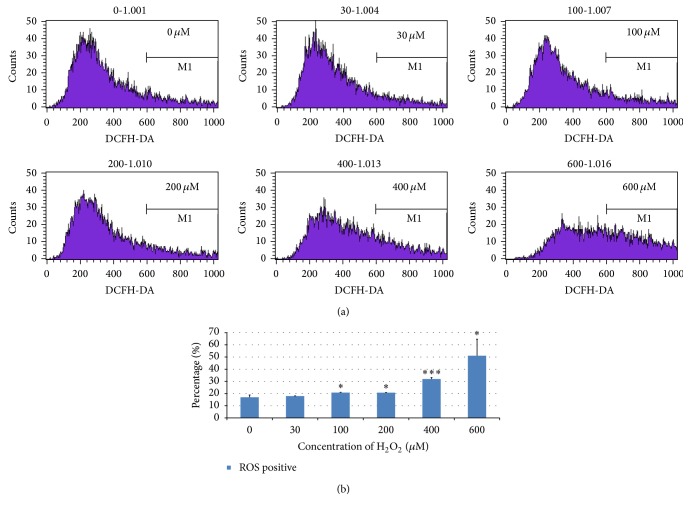
H_2_O_2_ treatment stimulates ROS in a dose-dependent manner. (a) The total ROS was determined through FCM assay. HepG2 cells were treated with H_2_O_2_ for 24 h. (b) The histogram shows the ROS positive cell percentage detected by FCM. ^*∗*^ *P* < 0.05; ^*∗∗∗*^ *P* < 0.001.

To confirm the ROS induction, FCM was used to detect the total ROS. As shown in Figure 4, 30 *μ*M of H_2_O_2_ did not increase ROS in HepG2 cells (*P* > 0.05). However, once the concentration of H_2_O_2_ was higher than 100 *μ*M, ROS levels were significantly increased (*P* < 0.05). These data showed that H_2_O_2_ could also stimulate ROS in a dose-dependent manner, which was consistent with the changes in cell biological functions.

### 3.4. Identification of miRNAs Modulated by Oxidative Stress

**Figure 5:**
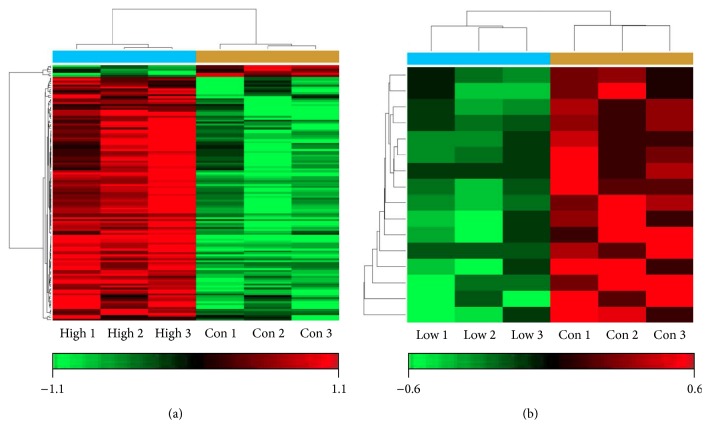
Changes in miRNAs expression profiles in H_2_O_2_ treated HepG2 cells. Total RNA was extracted from control group and HepG2 cells treated with 600 or 30 *μ*M H_2_O_2_ for 24 h. miRNAs microarray was performed as described in Materials and Methods. (a) High concentration versus control group, changes in miRNAs expression > 2-fold, and *Q* < 0.05 are illustrated by heat map. Green indicates a relatively low expression and red indicates a relatively high expression. (b) Low concentration versus control group, changes in miRNAs expression > 1.2-fold, and *Q* < 0.5 are illustrated by heat map. High: high concentration of H_2_O_2_ group, 600 *μ*M; Con: control group; Low: low concentration of H_2_O_2_ group, 30 *μ*M.

To verify whether miRNAs could be modulated by oxidative stress, we stimulated HepG2 cells with H_2_O_2_ to develop a cell model of oxidative stress. Taking into account the results listed above, we chose 30 and 600 *μ*M of H_2_O_2_ as low and high concentrations for miRNAs profiling, respectively. After stimulating HepG2 cells for 24 h, H_2_O_2_-induced changes in the miRNAs expression profiles were analyzed by the Affymetrix microRNA 4.0 Array, which contains 2578 probes and can interrogate all mature miRNAs sequences in miRBase Release 20. The results revealed that 131 miRNAs were deregulated in high concentration group under the condition of “*Q* < 0.05 and fold change > 2,” compared with normal control. Among them, 125 miRNAs were upregulated and 6 were downregulated (Figure 5(a)). However, there were no statistically significant differences between low concentration group and normal control under the same condition. After adjusting the condition to “*Q* < 0.5 and fold change > 1.2,” 16 deregulated miRNAs were determined in low concentration group, all of which were downregulated (Figure 5(b)). All of the deregulated miRNAs were listed in Supplementary Table 1 in Supplementary Material available online at http://dx.doi.org/10.1155/2016/7530853. Evidently, no deregulated miRNAs were overlapping between the two concentration groups. These data suggest that higher concentration of H_2_O_2_ mainly upregulated the expression of miRNAs, while lower concentration of H_2_O_2_ might have no effects and might even play the opposite role in the miRNAs expression. In view of no statistical differences between the low concentration group and normal control, we did not pursue the subsequent bioinformatics analysis of that group.

### 3.5. Identification of H_2_O_2_-Sensitive miRNAs Putative Target Genes and GO/Pathway Enrichment Analysis

Our study has identified 131 miRNAs that were significantly deregulated in response to 600 *μ*M H_2_O_2_, compared with normal control. As miRNAs play their biological roles through regulating target genes expression at the posttranscriptional level, we firstly predicted the target genes of H_2_O_2_-sensitive miRNAs by GCBI online tools, which are mainly based on the algorithms of miRanda and TargetScan. As a result, 13504 genes were predicted as putative target genes of high concentration H_2_O_2_-sensitive miRNAs.

**Figure 6:**
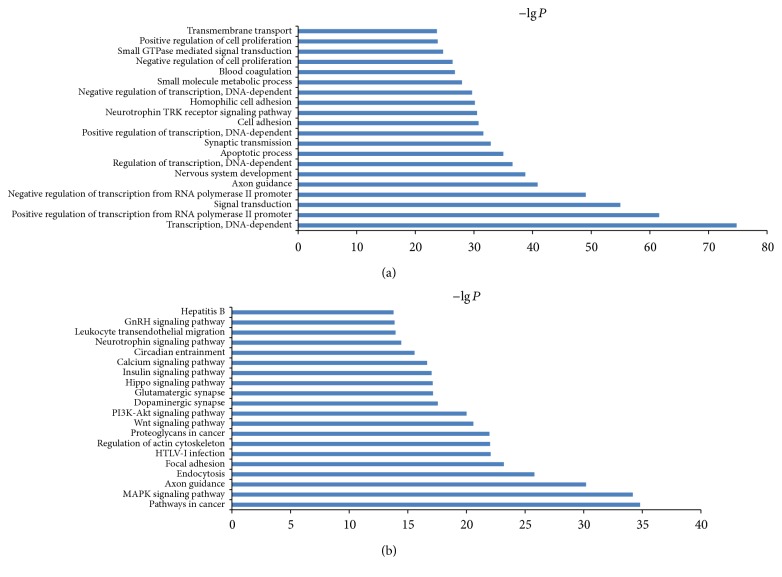
Significantly changed GO/pathways of predicted target genes of deregulated miRNAs after H_2_O_2_ treatment (600 *μ*M). (a) Significantly changed GOs of predicted target genes. The *y*-axis shows GO category and the *x*-axis shows −lg *P*. The larger −lg *P* indicated a smaller *P* value. (b) Significantly changed pathways of predicted target genes. The *y*-axis shows significantly changed pathways. −lg *P*: negative logarithm of the *P* value.

**Figure 7:**
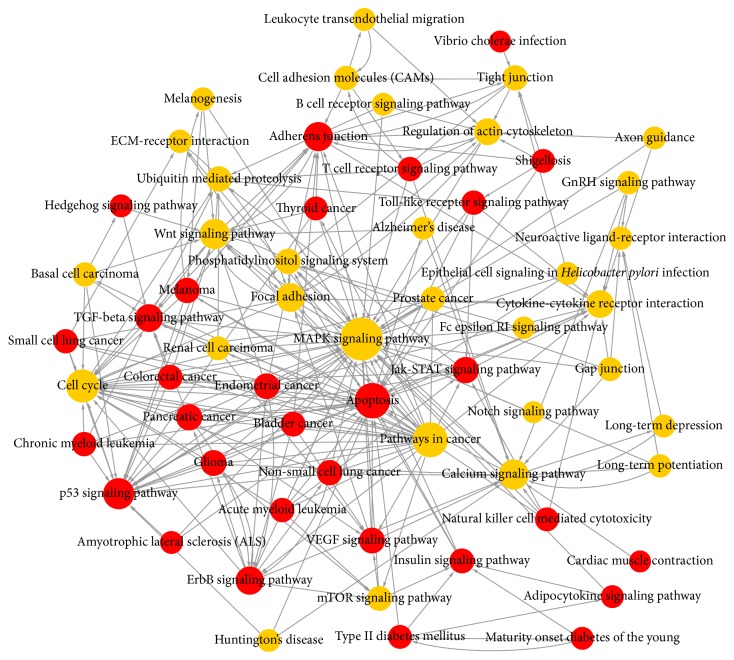
Pathway network (Path-net). Significantly changed pathways were connected in a Path-net to show the interaction network among these pathways. Each pathway in the network was measured by counting the upstream and downstream pathways. The red circle represents pathways involving upregulated miRNAs, while the yellow circle represents pathways involving both upregulated and downregulated miRNAs. The size of the circle represents the degree value and the lines show the interaction between pathways. A higher degree of pathway indicates that it plays a more important role in the signaling network.

To identify the biological functions of these genes, GO and pathway enrichment analysis were performed, respectively. As illustrated in Figure 6(a), the top ten deregulated GOs sensitive to high concentration of H_2_O_2_ were “transcription, DNA-dependent,” positive regulation of transcription from RNA polymerase II promoter, signal transduction, negative regulation of transcription from RNA polymerase II promoter, axon guidance, nervous system development, “regulation of transcription, DNA-dependent,” apoptotic process, and synaptic transmission. GO analysis obviously suggests that high concentration of H_2_O_2_ could affect expression of many miRNAs, through which many important functions such as transcription regulation, signal transduction, and apoptotic process are involved in apoptotic regulation of HepG2 cells. Combining with KEGG database, we analyzed the pathways in which the putative target genes were involved. As illustrated in Figure 6(b), the top ten deregulated pathways sensitive to high concentration of H_2_O_2_ were pathways in cancer, MAPK signaling pathway, axon guidance, endocytosis, focal adhesion, HTLV-I infection, regulation of actin cytoskeleton, proteoglycans in cancer, Wnt signaling pathway, and PI3K-Akt signaling pathway. Then, we performed Path-net analysis to draw an interaction network covering 61 significantly changed pathways (Figure 7). Among them, MAPK signaling pathway (degree = 44), apoptosis (degree = 29), pathways in cancer (degree = 28), and cell cycle (degree = 24) showed the highest degree, suggesting that these four pathways might play a core role in apoptosis induced by H_2_O_2_ treatment.

**Figure 8:**
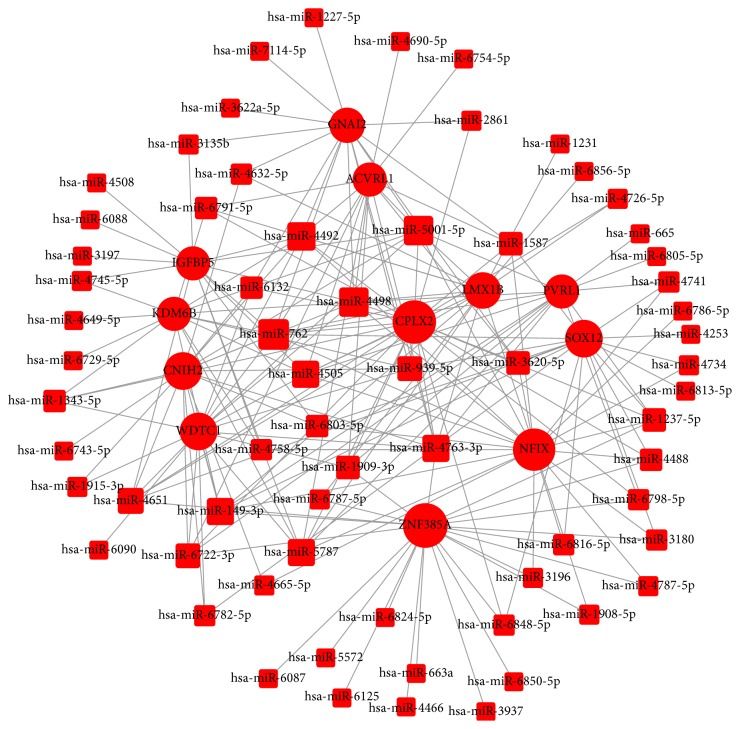
miRNAs-gene-network. According to the interactions between miRNAs and the intersected target genes, miRNAs-gene-network was constructed to illustrate the key regulatory functions of the identified miRNAs and their target genes. The red circles represent genes, while red square nodes represent upregulated miRNAs. The size of the circle or square node represents the degree value. A higher degree of gene/miRNAs indicates that it plays a more important role in the signaling network.

**Figure 9:**
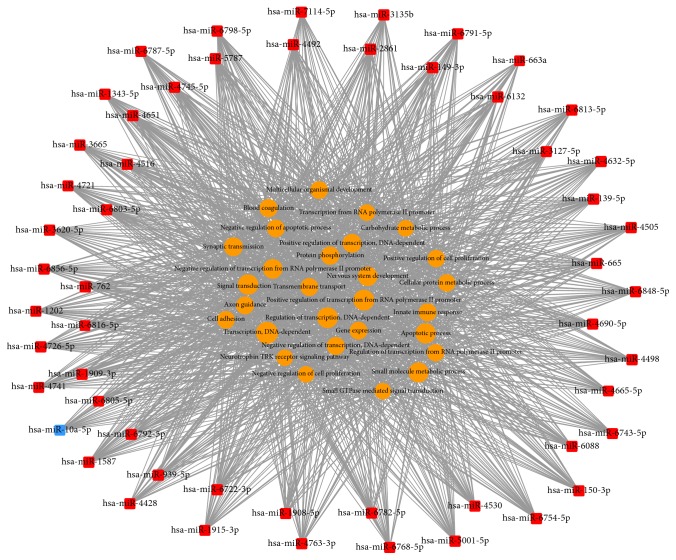
miRNAs-GO-network. The miRNAs-GO-network was generated according to the relationship of significant biological functions and miRNAs. The yellow circles represent GOs, red square nodes represent upregulated miRNAs, and blue square nodes represent downregulated miRNAs. The size of the circle or square node represents the degree value. A higher degree of GO/miRNAs indicates that it plays a more important role in the signaling network.

Based on the significantly regulated GOs and pathways, we selected intersected genes and further constructed miRNAs-gene-networks and miRNAs-GO-networks to screen the key regulatory functions of the identified miRNAs and their target genes, respectively. As shown in Figures 8 and 9 and Table 1, the top rated six miRNAs from the two analyses were the same, including hsa-miR-4763-3p, hsa-miR-149-3p, hsa-miR-762, hsa-miR-5001-5p, hsa-miR-5787, and hsa-miR-6791-5p. All of these miRNAs were upregulated by H_2_O_2_ treatment in HepG2 cells during apoptosis. The top six target genes were CPLX2 (complexin 2), ZNF385A (zinc finger protein 385A), NFIX (nuclear factor I/X (CCAAT-binding transcription factor)), CNIH2 (cornichon family AMPA receptor auxiliary protein 2), SOX12 (SRY- (sex determining region Y-) box 12), and WDTC1 (WD and tetratricopeptide repeats 1). Our analysis also showed that the deregulated miRNAs mainly play vital roles in various biological processes, including transcription regulation, apoptotic process, gene expression, and signal transduction. Taken together, these results represented comprehensive information on the biological function of H_2_O_2_ treatment in HepG2 cells. Through deregulation of certain miRNAs and several important pathways, elevated concentrations of H_2_O_2_ could decrease cell viability, inhibit cell proliferation, induce cell cycle arrest, and finally promote cell apoptosis in HepG2 cells.

## 4. Discussion

H_2_O_2_, one of the most studied ROS, is a protonated form of O_2_^−^ and can produce OH^•^ in the presence of transition metals like cupper or iron. H_2_O_2_ is also a hypochlorous acid precursor [17, 18]. As a soluble lipid, this strong oxidizing agent has been found to diffuse throughout the cell membrane via some aquaporins and can cause damage of cellular membranes, proteins, and DNA [19, 20]. Although H_2_O_2_ is widely used as oxidative stress stimuli, little is known about the biological functions of it on HepG2 cells. To investigate the role of H_2_O_2_ on HepG2 cells, we comprehensively examined the changes in cell biological functions, including cell viability, proliferation, cycle, and apoptosis. Specifically, H_2_O_2_ could significantly decrease cell viability and inhibit proliferation around 70.3 *μ*M, which was calculated as the IC50 value. Once the concentration of H_2_O_2_ is higher than 100 *μ*M, it can significantly induce cell cycle arrest in G2/M phase (*P* < 0.05). Meanwhile, if the concentration of H_2_O_2_ is higher than 200 *μ*M, it can obviously promote cell early apoptosis (*P* < 0.05). In contrast, HepG2 cells exhibit no statistically significant changes under the treatment of low concentration of H_2_O_2_. In general, H_2_O_2_ could decrease cell viability, inhibit cell proliferation, induce cell cycle arrest, and finally promote cell apoptosis in a dose-dependent manner. These results suggested that H_2_O_2 _may be used as an anticancer agent if used at appropriate concentrations [21, 22]. Indeed, many chemotherapy drugs play their roles in killing tumor cells by producing ROS [10]. It could be confirmed from another perspective that several clinical trials based on the concept that oxidants were toxic and antioxidants were favorable for cancer prevention were largely unsuccessful [23].

As mentioned above, DNA damage responses induced by H_2_O_2_ usually end up with the decrease of cell viability and promotion of apoptosis. In addition to its effects on apoptosis, H_2_O_2_ could also induce necrosis or autophagy depending on the stimulus intensity. Thus, H_2_O_2_ leads to cell death in different manners [24–27]. In this study, we found that 100 *μ*M H_2_O_2_ could decrease the cell viability to nearly 10% and induce obvious cell proliferation inhibition and cycle arrest, but there are no statistically significant differences in apoptosis under the same condition. This discrepancy might be caused by the following reasons. Firstly, different methodologies may lead to the inconsistent results. Specifically, cell viability and proliferation were detected by CCK-8 assay, while cell cycle and apoptosis were analyzed by FCM. Secondly, under the treatment of 100 *μ*M H_2_O_2_, nearly 90% reduction of cell viability was caused by multiple mechanisms, including cell apoptosis, necrosis, and autophagy. Finally, although H_2_O_2_ could induce biological changes in a dose-dependent manner, the potentially different sensitivity or reactivity of various biological functions, such as cell viability, cell proliferation, cell cycle, and cell apoptosis, might lead to the discrepancy. In other words, these biological functions react or adapt differently to 100 *μ*M H_2_O_2 _treatment.

Furthermore, to confirm the induction of ROS by H_2_O_2_ treatment, we used FCM to detect the total ROS. Our results showed that 100 *μ*M of H_2_O_2_ could significantly induce ROS (*P* < 0.05) and it also stimulates ROS in a dose-dependent manner. Although oxidative stress is closely related with liver cancer, further and detailed investigation is required to elucidate the molecular mechanisms of the interaction among oxidative stress, miRNAs, and liver cancer. By comparing the miRNAs expression profiles of control and high concentration group (600 *μ*M), we identified 131 differentially expressed miRNAs (*Q* < 0.05, fold change > 2), of which 125 were upregulated and 6 were downregulated (Figure 5(a)). However, only 16 downregulated miRNAs could be determined in low concentration group after adjusting the condition to “*Q* < 0.5 and fold change > 1.2” (Figure 5(b)). These data suggest that higher concentration of H_2_O_2_ mainly upregulated the expression of miRNAs, while lower concentration of H_2_O_2_ might play the opposite role on the miRNAs expression. In view of the fact that miRNAs generally inhibit translation of their target genes, we could easily deduce that higher concentration of H_2_O_2_ mainly downregulates the translation of target proteins, while lower concentration of H_2_O_2_ might play the opposite role. Among the top ten fold changed miRNAs (Supplementary Table 1), half of them (hsa-miR-371b-5p, hsa-miR-663a, hsa-miR-1225-5p, hsa-miR-1202, and hsa-miR-572) were closely linked to the occurrence and development of tumors and might play vital roles as oncogenes or tumor suppressor genes [28–31]. These data confirmed that oxidative stress could interplay with cancer biology through modulating specific miRNAs expression. However, the rest of the top changed miRNAs were relatively novel miRNAs, which are rarely studied before and their functions are mostly unknown.

Then, 13504 genes were predicted as putative target genes of high concentration H_2_O_2_-sensitive miRNAs using target prediction methods. According to the result of GO analysis, the predicted target genes were mainly involved in transcription, signal transduction, regulation of transcription, apoptotic process, and synaptic transmission (Figure 6(a)). These biological processes are reported to be crucial in the regulation of apoptosis. As for biological pathways, pathways in cancer, MAPK signaling pathway, endocytosis, focal adhesion, proteoglycans in cancer, Wnt signaling pathway, and PI3K-Akt signaling pathway were the top enriched pathways of the predicted target genes (Figure 6(b)). It is well known that these pathways play important roles in regulation of cell apoptosis and survival outcome. The Path-net analysis covering 61 significantly changed pathways showed that MAPK signaling pathway, apoptosis, pathways in cancer, and cell cycle play a more important role in apoptosis induced by H_2_O_2_ treatment (Figure 7). To screen for the important genes involved in apoptosis regulated by H_2_O_2_ in HepG2 cells, we performed regulatory network analysis by overlapping the significantly regulated miRNAs, GOs, and pathways. The results showed that the top six miRNAs were hsa-miR-4763-3p, hsa-miR-149-3p, hsa-miR-762, hsa-miR-5001-5p, hsa-miR-5787, and hsa-miR-6791-5p, while the top six target genes were CPLX2, ZNF385A, NFIX, CNIH2, SOX12, and WDTC1 (Figures 8 and 9). Taken together, these data suggest that H_2_O_2_ treatment may regulate biological functions of HepG2 cells through the changes in expression of specific miRNAs.

However, it is well acknowledged that predicting the miRNAs targets merely by the means of bioinformatics is not sufficient. miRNAs targets should be strictly verified by further functional experiments, such as gain or loss of miRNAs function, luciferase report assay, and western blot confirmation [32, 33]. Therefore, our future investigations will be aimed at picking up some significantly deregulated miRNAs and carrying out experiments to confirm their targets and then unveil the mechanisms involved in the interplay among oxidative stress, miRNAs, and cancer.

## 5. Conclusions

Since H_2_O_2_ could decrease cell viability, inhibit proliferation, induce cycle arrest, and promote apoptosis in a dose-dependent manner, this ROS is a very promising potential therapeutic tool to fight cancer. The proper and cautious use of H_2_O_2_ in combination with significantly deregulated miRNAs' mimics or antagomirs may have synergistic effects on increasing liver cancer cell death.

## Supplementary Material

To verify whether miRNAs could be modulated by oxidative stress, we utilize miRNAs array to profile their expression changes after H_2_O_2_ treatment of 30 and 600 μM, respectively. The results revealed that 131 miRNAs were deregulated in high concentration group under the condition of “Q < 0.05 and Fold change > 2”, compared with normal control. Among them, 125 miRNAs were upregulated and 6 were downregulated. However, there were no statistically significant differences between low concentration group and normal control under the same condition. After adjusting the condition to “Q < 0.5 and Fold change >1.2”, 16 deregulated miRNAs were determined in low concentration group, all of which were downregulated.

## Figures and Tables

**Table 1:** The top 10 miRNAs with high degrees of miRNAs-gene-networks and miRNAs-GO-networks.

Rank	miRNAs-gene-networks			miRNAs-GO-networks		
	miRNAs	Degree	Feature	miRNAs	Degree	Feature
1	hsa-miR-4763-3p	265	Up	hsa-miR-4763-3p	436	Up
2	hsa-miR-149-3p	251	Up	hsa-miR-149-3p	408	Up
3	hsa-miR-762	211	Up	hsa-miR-762	346	Up
4	hsa-miR-5001-5p	207	Up	hsa-miR-6791-5p	337	Up
5	hsa-miR-5787	195	Up	hsa-miR-5001-5p	336	Up
6	hsa-miR-6791-5p	188	Up	hsa-miR-5787	333	Up
7	hsa-miR-4498	180	Up	hsa-miR-2861	332	Up
8	hsa-miR-4651	164	Up	hsa-miR-4505	322	Up
9	hsa-miR-4505	159	Up	hsa-miR-4498	320	Up
10	hsa-miR-4632-5p	150	Up	hsa-miR-665	303	Up
