# Supplementary material for: Identification of MicroRNAs Involved in Growth Arrest and Apoptosis in Hydrogen Peroxide-Treated Human Hepatocellular Carcinoma Cell Line HepG2
Source: Oxid Med Cell Longev. 2016 Aug 15;2016:7530853. doi: 10.1155/2016/7530853 (PMC5002491; doi:10.1155/2016/7530853)
Supplement: Supplementary file 1 — To verify whether miRNAs could be modulated by oxidative stress, we utilize miRNAs array to profile their expression changes after H2O2 treatment of 30 and 600 μM, respectively. The results revealed that 131 miRNAs were deregulated in high concentration group under the condition of “Q < 0.05 and Fold change > 2”, compared with normal control. Among them, 125 miRNAs were upregulated and 6 were downregulated. However, there were no statistically significant differences between low concentration group and normal control under the same condition. After adjusting the condition to “Q < 0.5 and Fold change >1.2”, 16 deregulated miRNAs were determined in low concentration group, all of which were downregulated. [file 7530853.f1.docx]

**Supplementary Tables**

**Supplementary Table 1. Up- and down-regulated miRNAs in H_2_O_2_ treated HepG2 cells.**

| 1. **High concentration (600 μM) versus control group. (Q < 0.05, Fold change > 2)** | | | | | | | | | | | |
| --- | --- | --- | --- | --- | --- | --- | --- | --- | --- | --- | --- |
| **miRNA** | **Fold change** | **Feature** | **miRNA** | **Fold change** | **Feature** | **miRNA** | **Fold change** | **Feature** | **miRNA** | **Fold change** | **Feature** |
| hsa-miR-371b-5p | 7.348477 | up | hsa-miR-6068 | 7.089294 | up | hsa-miR-663a | 6.725608 | up | hsa-miR-6820-5p | 6.237283 | up |
| hsa-miR-1225-5p | 6.088823 | up | hsa-miR-1202 | 6.018439 | up | hsa-miR-6813-5p | 5.990627 | up | hsa-miR-572 | 5.629193 | up |
| hsa-miR-3197 | 5.028351 | up | hsa-miR-4492 | 5.023914 | up | hsa-miR-3180 | 4.972522 | up | hsa-miR-4508 | 4.842646 | up |
| hsa-miR-6132 | 4.835348 | up | hsa-miR-6848-5p | 4.623262 | up | hsa-miR-4505 | 4.599607 | up | hsa-miR-3621 | 4.340172 | up |
| hsa-miR-6126 | 4.278259 | up | hsa-miR-6075 | 4.250213 | up | hsa-miR-4530 | 4.198824 | up | hsa-miR-4734 | 4.074539 | up |
| hsa-miR-4532 | 3.946948 | up | hsa-miR-6768-5p | 3.911165 | up | hsa-miR-1909-3p | 3.833301 | up | hsa-miR-4466 | 3.796487 | up |
| hsa-miR-3185 | 3.781577 | up | hsa-miR-2861 | 3.776816 | up | hsa-miR-1915-3p | 3.774927 | up | hsa-miR-6742-5p | 3.756872 | up |
| hsa-miR-4674 | 3.66685 | up | hsa-miR-6789-5p | 3.635754 | up | hsa-miR-4417 | 3.624982 | up | hsa-miR-8069 | 3.623957 | up |
| hsa-miR-7108-5p | 3.587856 | up | hsa-miR-4488 | 3.560815 | up | hsa-miR-1469 | 3.542843 | up | hsa-miR-1587 | 3.523945 | up |
| hsa-miR-4507 | 3.394821 | up | hsa-miR-4485 | 3.311537 | up | hsa-miR-638 | 3.280752 | up | hsa-miR-762 | 3.241566 | up |
| hsa-miR-3940-5p | 3.217361 | up | hsa-miR-6805-5p | 3.205835 | up | hsa-miR-4651 | 3.145631 | up | hsa-miR-4707-5p | 3.126016 | up |
| hsa-miR-1237-5p | 3.109253 | up | hsa-miR-3620-5p | 3.092557 | up | hsa-miR-7704 | 3.074193 | up | hsa-miR-4484 | 3.06888 | up |
| hsa-miR-4745-5p | 3.063936 | up | hsa-miR-4634 | 3.040358 | up | hsa-miR-4632-5p | 2.99682 | up | hsa-miR-6727-5p | 2.907574 | up |
| hsa-miR-5787 | 2.894433 | up | hsa-miR-1227-5p | 2.853645 | up | hsa-miR-3135b | 2.802132 | up | hsa-miR-4463 | 2.798242 | up |
| hsa-miR-3196 | 2.796944 | up | hsa-miR-3656 | 2.757645 | up | hsa-miR-6729-5p | 2.757295 | up | hsa-miR-6125 | 2.756277 | up |
| hsa-miR-149-3p | 2.733141 | up | hsa-miR-4497 | 2.73032 | up | hsa-miR-6790-5p | 2.721106 | up | hsa-miR-665 | 2.711938 | up |
| hsa-miR-4787-5p | 2.706122 | up | hsa-miR-4516 | 2.671012 | up | hsa-miR-6798-5p | 2.643372 | up | hsa-miR-6722-3p | 2.593723 | up |
| hsa-miR-7114-5p | 2.587864 | up | hsa-miR-6816-5p | 2.565665 | up | hsa-miR-6803-5p | 2.514878 | up | hsa-miR-6754-5p | 2.496822 | up |
| hsa-miR-4649-5p | 2.478214 | up | hsa-miR-1343-5p | 2.469474 | up | hsa-miR-3917 | 2.464956 | up | hsa-miR-4253 | 2.423614 | up |
| hsa-miR-6088 | 2.415229 | up | hsa-miR-4758-5p | 2.397139 | up | hsa-miR-939-5p | 2.368212 | up | hsa-miR-6743-5p | 2.346131 | up |
| hsa-miR-4486 | 2.318211 | up | hsa-miR-6824-5p | 2.289561 | up | hsa-miR-4690-5p | 2.263884 | up | hsa-miR-4498 | 2.261421 | up |
| hsa-miR-1908-5p | 2.245914 | up | hsa-miR-1228-5p | 2.178449 | up | hsa-miR-6087 | 2.112768 | up | hsa-miR-6786-5p | 2.068943 | up |
| hsa-miR-4741 | 4.10796 | up | hsa-miR-6724-5p | 3.893599 | up | hsa-miR-6802-5p | 3.845518 | up | hsa-miR-4726-5p | 3.598384 | up |
| hsa-miR-3937 | 3.419596 | up | hsa-miR-150-3p | 3.264434 | up | hsa-miR-4467 | 3.195074 | up | hsa-miR-6800-5p | 3.075666 | up |
| hsa-miR-139-5p | 2.978479 | up | hsa-miR-6791-5p | 2.882374 | up | hsa-miR-6821-5p | 2.859674 | up | hsa-miR-1231 | 2.606078 | up |
| hsa-miR-5001-5p | 2.507648 | up | hsa-miR-4763-3p | 2.504473 | up | hsa-miR-4665-5p | 2.453938 | up | hsa-miR-6861-5p | 2.429962 | up |
| hsa-miR-3648 | 2.414096 | up | hsa-miR-1246 | 2.389179 | up | hsa-miR-6089 | 2.387594 | up | hsa-miR-6856-5p | 2.27876 | up |
| hsa-miR-3665 | 2.24792 | up | hsa-miR-3187-3p | 2.233004 | up | hsa-miR-135a-3p | 2.158777 | up | hsa-miR-6090 | 2.134893 | up |
| hsa-miR-8063 | 3.049769 | up | hsa-miR-5572 | 2.637084 | up | hsa-miR-3127-5p | 2.577368 | up | hsa-miR-6850-5p | 2.333896 | up |
| hsa-miR-6782-5p | 2.218585 | up | hsa-miR-3622a-5p | 2.180865 | up | hsa-miR-6787-5p | 2.159218 | up | hsa-miR-6792-5p | 2.373032 | up |
| hsa-miR-4721 | 2.197885 | up | hsa-miR-4750-5p | 2.164756 | up | hsa-miR-6890-5p | 2.127968 | up | hsa-miR-4706 | 2.107374 | up |
| hsa-miR-1226-3p | -4.544148 | down | hsa-miR-1247-5p | -2.880127 | down | hsa-miR-1303 | -2.152835 | down | hsa-miR-33b-3p | -2.033996 | down |
| hsa-miR-4428 | 2.261505 | up | hsa-miR-22-5p | -2.066546 | down | hsa-miR-10a-5p | -2.016948 | down |  |  |  |
| 1. **Low concentration (30 μM) versus control group. (Q < 0.5, Fold change > 1.2)** | | | | | | | | | | | |
| **miRNA** | **Fold change** | **Feature** | **miRNA** | **Fold change** | **Feature** | **miRNA** | **Fold change** | **Feature** | **miRNA** | **Fold change** | **Feature** |
| hsa-miR-6133 | -2.253309 | down | hsa-miR-1184 | -2.245369 | down | hsa-miR-7111-5p | -2.18579 | down | hsa-miR-8085 | -1.971213 | down |
| hsa-miR-6833-5p | -1.874466 | down | hsa-miR-6893-5p | -1.730764 | down | hsa-miR-6877-5p | -1.616255 | down | hsa-miR-6870-5p | -1.59031 | down |
| hsa-miR-148a-5p | -1.566222 | down | hsa-miR-483-5p | -1.515302 | down | hsa-miR-4710 | -1.490756 | down | hsa-miR-98-5p | -1.48285 | down |
| hsa-miR-132-5p | -1.440816 | down | hsa-miR-3622a-5p | -1.43222 | down | hsa-miR-378e | -1.343756 | down | hsa-miR-489-3p | -1.338286 | down |
